# Analysing the visible conformational substates of the FK506-binding protein FKBP12

**DOI:** 10.1042/BJ20130276

**Published:** 2013-07-12

**Authors:** Sourajit M. Mustafi, Hui Chen, Hongmin Li, David M. LeMaster, Griselda Hernández

**Affiliations:** *Wadsworth Center, New York State Department of Health, Empire State Plaza, Albany, NY 12201, U.S.A.; †Department of Biomedical Sciences, School of Public Health, University at Albany - SUNY, Empire State Plaza, Albany, NY 12201, U.S.A.

**Keywords:** conformational dynamics, FK506-binding protein of 12 kDa (FKBP12), NMR, slow exchange, X-ray structure, CPMG, Carr–Purcell–Meiboom–Gill, CT-HSQC, constant-time heteronuclear single-quantum coherence, FKBP12, FK506-binding protein of 12 kDa, mTOR, mammalian target of rapamycin, NOE, nuclear Overhauser effect, RyR, ryanodine receptor, TCEP, tris-(2-carboxyethyl)phosphine

## Abstract

The ^1^H-^15^N 2D NMR correlation spectrum of the widely studied FK506-binding protein FKBP12 (FK506-binding protein of 12 kDa) contains previously unreported peak doublings for at least 31 residues that arise from a minor conformational state (12% of total) which exchanges with the major conformation with a time constant of 3.0 s at 43°C. The largest differences in chemical shift occur for the 80′s loop that forms critical recognition interactions with many of the protein partners for the FKBP family. The residues exhibiting doubling extend into the adjacent strands of the β-sheet, across the active site to the α-helix and into the 50′s loop. Each of the seven proline residues adopts a *trans*-peptide linkage in both the major and minor conformations, indicating that this slow transition is not the result of prolyl isomerization. Many of the residues exhibiting resonance doubling also participate in conformational line-broadening transition(s) that occur ~10^5^-fold more rapidly, proposed previously to arise from a single global process. The 1.70 Å (1 Å=0.1 nm) resolution X-ray structure of the H87V variant is strikingly similar to that of FKBP12, yet this substitution quenches the slow conformational transition throughout the protein while quenching the line-broadening transition for residues near the 80′s loop. Line-broadening was also decreased for the residues in the α-helix and 50′s loop, whereas line-broadening in the 40′s loop was unaffected. The K44V mutation selectively reduces the line-broadening in the 40′s loop, verifying that at least three distinct conformational transitions underlie the line-broadening processes of FKBP12.

## INTRODUCTION

The immunophilin FKBP12 (FK506-binding protein of 12 kDa) was first characterized for its role in binding the immunosuppressants FK506 and rapamycin. The FK506-bound FKBP12 forms a ternary complex with calcineurin that inhibits the protein phosphatase activity of that enzyme which, in turn, blocks a key T-cell activation pathway involved in tissue transplant rejection [1]. In complex with rapamycin, FKBP12 binds to and inhibits the protein kinase mTOR (mammalian target of rapamycin) [2]. The major role of mTOR in regulating cell growth and cancer progression has greatly stimulated the analysis of this interaction with the rapamycin–FKBP12 complex.

Biochemical isolation of the RyR (ryanodine receptor) Ca^2+^ channels from skeletal muscle demonstrated that four molecules of FKBP12 are bound to each tetrameric RyR1 receptor [3]. Analogous isolation of the cardiac ryanodine receptor RyR2 revealed the binding of the highly homologous FKBP12.6 protein [4]. Although most subsequent studies have been interpreted in terms of distinct regulation of skeletal muscle RyR1 by FKBP12 and cardiac muscle RyR2 regulation by FKBP12.6, a number of studies have pointed to a more complex pattern of interactions. FKBP12 germline knockout mice die *in utero* due to severe cardiac ventricular defects [5], although mouse cardiomyocyte-specific conditional gene knockout studies appear to indicate a non-cardiomyocyte origin for these ventricular defects [6]. Nevertheless, cardiomyocyte-restricted overexpression of FKBP12 results in cardiac pathologies leading to a high incidence of sudden death [6]. Recent studies point to a more substantial and partially antagonistic role for FKBP12-mediated calcium regulation in cardiac muscle [7].

The peptidylprolyl isomerase activity of FKBP12 appears to play a more central role in its involvement in neurodegeneration arising from protein aggregation pathologies. Not only is FKBP12 found along with the aggregated α-synuclein in the Lewy body deposits of Parkinson's disease patients [8], but also it accelerates the aggregation of α-synuclein *in vitro* [9] and *in vivo* [10]. The accumulation of FKBP12 in the neurofibrillary tangles of Alzheimer's disease suggests an analogous binding to the conformationally disordered tau protein [11]. Similarly, the binding of FKBP12 to the amyloid precursor protein, confirmed by both yeast two-hybrid and co-immunoprecipitation [11,12], is selectively disrupted by addition of the FK506 inhibitor.

Continuing interest in FKBP12 as a target for clinical therapies is indicated by the deposition of over 30 X-ray structures of the wild-type and mutational variants of the human protein both free and bound to either small-molecule inhibitors or in physiological protein–protein complexes. Generally, these structures have indicated that only modest changes in the conformation of FKBP12 are induced by these binding interactions. On the other hand, NMR relaxation studies have demonstrated that a substantial number of residues of the unligated protein undergo conformational transition(s) in the microsecond–millisecond timeframe which gives rise to a characteristic line-broadening for the resonances of the neighbouring atoms. Brath, Akke, Yang, Kay and Mulder [13] reported ^13^C relaxation dispersion measurements of FKBP12 that identified 12 exchange-broadened methyl resonances which they interpreted as representing a single global conformational exchange process with a time constant of ~130 μs. On the basis of the fact that many of these methyl-bearing residues line the active site and correspond to positions that undergo changes in chemical shift upon the binding of FK506 or rapamycin, the authors proposed that these conformational dynamics are functionally significant. Subsequently, Brath and Akke [14] carried out an analogous ^15^N relaxation dispersion study to characterize conformational dynamics in the backbone of FKBP12. The 23 amides that exhibited conformational exchange primarily lay within the backbone segments of residues 26–44, 53–57 and 75–98 which when combined with their earlier ^13^C measurements were fitted to a single global time constant of 120 μs.

These line-broadening effects arise from individual nuclei rapidly transferring between conformations that exhibit different ^15^N (or ^13^C) chemical shifts. Although the minor conformations do not give rise to distinct observable resonances, when the exchange rates and populations are within suitable ranges, the chemical shifts for the minor conformation can be inferred [15]. Brath and Akke [14] argued that their proposed collective transition corresponds to the catalytic transition state conformation of the enzyme, despite the fact that they observed an absence of any correlation between the magnitude of the ligand-induced shifts and the resonance line-broadening for the various affected residues. Their observation that the binding of FK506 quenches the conformational exchange broadening of the backbone resonances was subsequently verified by Sapienza, Mauldin and Lee [16]. More surprisingly, the latter authors found that, although rapamycin also suppressed the conformational exchange broadening of the backbone resonances in the residue segments 53–57 and 75–98, the exchange broadening for the 26–44 segment becomes more extensive, extending back to residue 23.

To gain further insight into the conformational processes exhibited by FKBP12, we have applied a combination of NMR measurements, crystallographic analysis and structure-based mutagenesis.

## EXPERIMENTAL

### Protein preparation

Genes for the wild-type as well as C22V, C22V+H87F, C22V+H87V and C22V+K44V variants of human FK506-binding protein were chemically synthesized (Genscript) from the wild-type gene sequence, with codon optimization for expression in *Escherichia coli*. The gene for the C48L variant of the (*Saccharomyces cerevisiae*) FKBP12 sequence as given in the crystal structure [17] was similarly produced. The genes were cloned into the expression vector pET11a and then transformed into the BL21(DE3) strain (Novagen) for expression. All expression strains were grown in minimal medium containing 0.1% ^15^NH_4_Cl as a nitrogen source, as described previously [18]. For U-^13^C,^15^N-enriched samples, 0.2% [U-^13^C]glucose (Cambridge Isotopes) was substituted for the unlabelled glucose used for preparing the U-^15^N samples. The U-^2^H,^15^N-enriched samples were prepared using [U-^2^H]glycerol (Cambridge Isotopes) as carbon source in a ^2^H_2_O-containing minimal medium as described previously [18].

The cells were grown at 37°C to a *D*_600_ of 0.5 before cooling to 25°C and then inducing with 0.5 mM IPTG to a *D*_600_ of 0.7. Following overnight agitation at 25°C, the cells were pelleted by centrifugation and frozen at −80°C. Cell lysis and ammonium sulfate fractionation followed by Sephadex G-50 gel exclusion and SP Sepharose FF ion-exchange chromatography were carried out as described previously [19]. For the purification of wild-type FKBP12, 1 mM DTT was added to all buffers.

Following purification, solid Tris base was added to the U-^2^H,^15^N-enriched protein samples to obtain a pH above 9, and the samples were incubated at 25°C for at least 3 h to back-exchange the slowly exchanging amide sites. For wild-type FKBP12, 1 mM TCEP [tris-(2-carboxyethyl)phosphine] was added for this exchange step. All protein samples were concentrated via centrifugal ultrafiltration and then equilibrated into a pH 6.5 buffer containing 25 mM sodium phosphate (with 2 mM DTT and 2 mM TCEP for the wild-type protein) by a series of centrifugal concentration steps. A final protein concentration of 1 mM was used for the NMR relaxation experiments. For the crystallization trials, the H87V protein sample was neutralized and then equilibrated into 5 mM sodium chloride and concentrated by centrifugal ultrafiltration.

### NMR spectroscopy

NMR data were collected on a Bruker Avance III 600 MHz spectrometer, a Bruker Avance II 800 MHz spectrometer and a Bruker Avance II 900 MHz spectrometer at 25°C. Backbone resonance assignments were carried out using standard HNCO [20], HN(CA)CO [20], HNCACB [21] and HN(CO)CACB [22] experiments. Side-chain resonance assignments utilized 2D CT-HSQC (constant-time heteronuclear single-quantum coherence) [23], 3D HCCH-TOCSY [24] and 3D HCCCONH [25,26] experiments. Chemical shift assignments for the major and minor slow exchange states have been deposited in the BMRB (Biological Magnetic Resonance Bank) under accession numbers 19240 and 19241 respectively. Minor adaptations of standard T_1_, T_2_ and heteronuclear NOE (nuclear Overhauser effect) experiments [27] were utilized. The NOE sequence incorporated the optimized ^1^H saturation protocol proposed by Ferrage, Cowburn, Ghose and colleagues [28]. T_1_ relaxation delay periods of 0.05, 0.15, 0.25, 0.40, 0.15, 0.60, 0.85, 1.20, 0.60 and 1.60 s were used. To minimize ^15^N offset effects on the derived T_2_ values, measurements for CPMG (Carr–Purcell–Meiboom–Gill) periods of 17, 34, 51, 68, 85 and 102 ms were repeated at equally spaced ^15^N carrier frequencies (four sets at 600 and six sets at 800). For each resonance, the fitted exponentials for each set were then averaged according to the ^15^N offset using a linear weighting varying from 1.0 on resonance to 0.0 at an offset equal to 15% of the ^15^N B_1_ field strength for the CPMG pulses. Error estimates for the NOE measurements were obtained from the difference between two independent datasets. Uncertainties for the T_1_ time constants were derived from covariance analysis of the fitted exponential (Origin 8.6, OriginLab). T_2_ error estimation for each resonance lying within the range of carrier frequencies used in these measurements was derived from the set of fitted exponentials used to obtain the individual averaged T_2_ values. FELIX software (Felix NMR) was used for NMR data processing, and Fast Modelfree software [29] was used for NMR relaxation analysis.

### X-ray crystallography

Crystals of the H87V protein were grown at room temperature (22°C) in hanging drops, by mixing 2 μl of protein solution at 21.5 mg/ml concentration with an equal volume of reservoir solution containing 1.7 M sodium malonic acid, 0.1 M Hepes (pH 7.4) and 5% 2-methyl-2,4-pentanediol. The crystals belong to space group *C*_2_ with cell parameters *a*=70.55 Å (1 Å=0.1 nm), *b*=35.95 Å, *c*=40.92 Å and β=95.89°. There is one molecule per asymmetric unit, with a crystal solvent content of 48%. Before data collection, crystals were gradually transferred to a reservoir solution containing a higher concentration of malonic acid up to 80% at 5% per step, and then flash-cooled under a nitrogen stream at 100 K, and stored in liquid nitrogen. Diffraction data were collected at 100 K using an RAxisIV++ detector and an in-house Rigaku microfocus MicroMax-007 X-ray generator. All of the data were processed and scaled using CrystalClear 1.3.6 software (Rigaku). With the high-resolution structure of FKBP12 (PDB code 2PPN [30]) used as a search model, clear solutions were found with the PHASER molecular replacement program within the PHENIX suite [31]. Structural refinement was carried out using PHENIX. Model rebuilding was carried out using Coot [32]. Figures of crystallographic structures were generated using Chimera software [33].

## RESULTS AND DISCUSSION

### Extensive NMR resonance doubling in FKBP12 indicative of a slow conformational transition

Previously, we [19] reported amide hydrogen exchange measurements on human FKBP12 in demonstrating that this experimental monitor of the thermodynamic acidity of the backbone peptide groups is acutely sensitive to protein conformation [34]. As we have shown for FKBP12 and several other well-characterized proteins, the amide hydrogen exchange rate data are reasonably robustly predictable from full atom structural models by continuum dielectric methods, and this analysis provides a valuable characterization of the conformational distribution of the protein native state [35–37]. Since these hydrogen exchange measurements utilize mildly basic conditions for extended time periods, we substituted valine for Cys^22^ of wild-type FKBP12 to circumvent the chemical modifications that can occur at cysteine residues under such conditions. Val^22^ is found throughout lower eukaryotes with the cysteine substitution not appearing until during the evolution of fish, not long before the divergence of the FKBP12 and FKBP12.6 subfamilies [38]. Model building from the high-resolution X-ray structure of human FKBP12 [30] indicated an internal cavity which readily accommodates the second methyl group of valine without apparent steric conflict.

**Figure 1:**
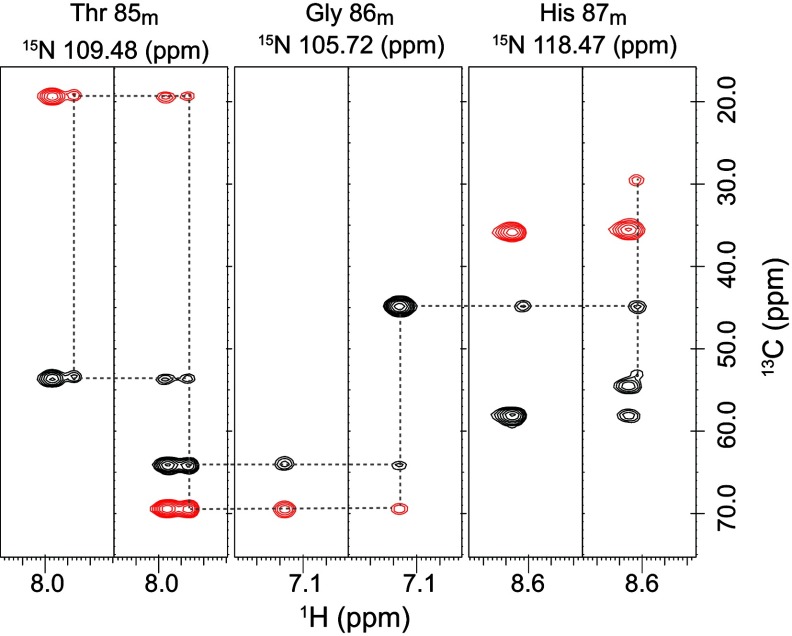
Backbone resonance assignment of the minor slow exchange conformation in the C22V variant of human FKBP12 Strips from the HN(CO)CACB (left) and HNCACB (right) datasets at the ^15^N frequencies for the amide resonances of the minor slow exchange conformation are illustrated for the sequence from Thr^85^ to His^87^. The C^α^ resonances are phased to yield positive (black) cross-peaks, whereas the C^β^ cross-peaks are negative (red).

During the course of our NMR experiments on the C22V variant of FKBP12, we noted a substantial set of minor peaks at approximately 15% of the intensities for the major amide resonances in the standard ^1^H-^15^N 2D correlation spectrum. MS analysis indicated a single chemical species, and backbone resonance assignment experiments on a U-^13^C,^15^N-labelled sample enabled us to establish the sequential connectivities for these minor cross-peaks, as illustrated for the residue sequence Thr^85^–His^87^ (Figure 1). This evidence for extensive doubling of the amide resonances was quite surprising given that such an effect has been unreported in not only each of the relaxation studies discussed above, but also for a number of other publications presenting ^1^H-^15^N 2D correlation spectra of the unligated wild-type FKBP12 in which no such minor peaks are indicated [39–44]. On the other hand, two publications have presented ^1^H-^15^N 2D correlation plots of FKBP12 at apparently lower contour levels that indicate a pattern of minor peaks [45,46], although no discussion of this heterogeneity was offered. On the other hand, the initial resonance assignment study by Rosen et al. [39] reported peak doubling for the H^α^ of Ala^84^ and for one of the two H^α^ peaks of Gly^58^. During the course of their histidine pH titration study, Yu and Fesik [47] also reported a doubling for the H^ϵ^-C^ϵ^ cross-peak of His^87^.

**Figure 2:**
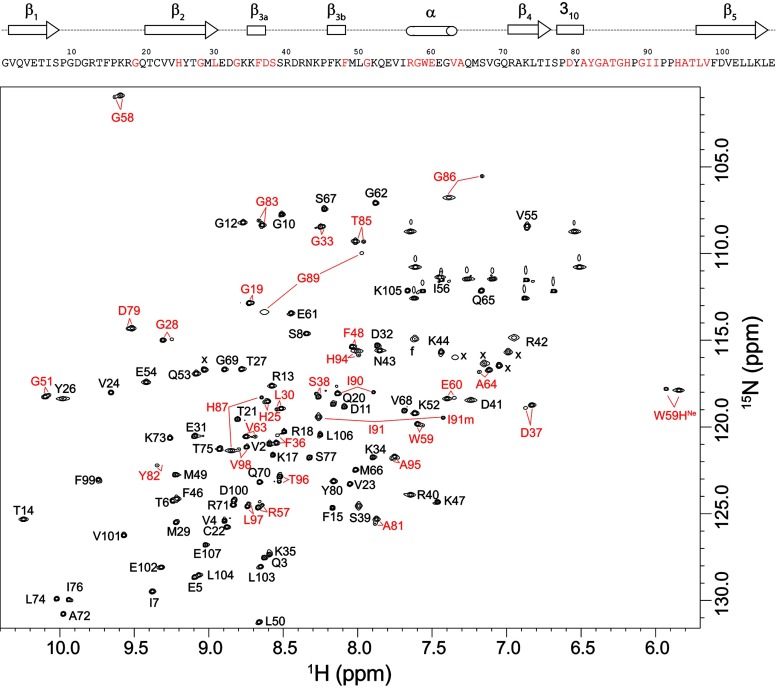
^1^H-^15^N 2D NMR correlation spectrum of U-^2^H,^15^N-enriched wild-type FKBP12 Residues exhibiting resolved resonances for the minor slow exchange conformation are indicated in red. Ala^84^ and Gly^89^ exhibit the most rapid amide hydrogen exchange in the protein [19], resulting in severe broadening in the ^1^H dimension at pH 6.5. These resonances are readily observed at pH 5.5. The severely broadened resonance for the major conformation of Tyr^82^ is visible at a lower contour level. The hydrogen exchange rate for this residue is too low to contribute to line-broadening. Folded side-chain resonances are indicated with x.

**Figure 3:**
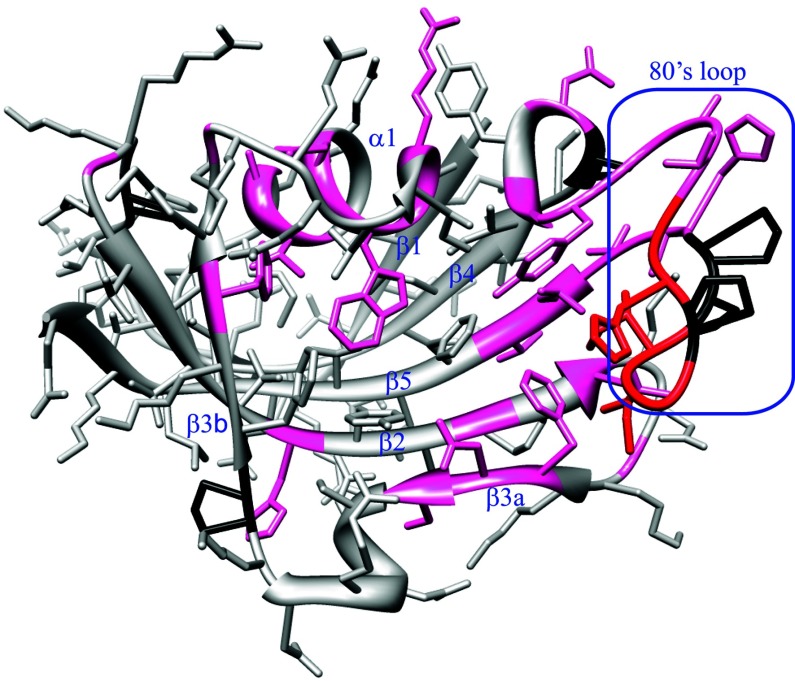
Structural distribution of residues exhibiting amide resonance doubling due to slow conformational exchange in FKBP12 Residues that yield doublings of their amide resonances separated by more than 0.15 p.p.m. (averaged as Δ^1^H and 0.2×Δ^15^N [63]) are indicated in red. Residues exhibiting smaller chemical shift differences between the two conformational states are indicated in magenta. Proline residues are marked in black.

To gain further insight into this issue, we prepared a U-^2^H,^15^N-enriched sample of wild-type FKBP12 which yielded a well-resolved ^1^H-^15^N 2D correlation spectrum in which 31 of the 99 backbone amides and the indole side chain of Trp^59^ give rise to both major and minor resonances (Figure 2). Excluding significantly overlapped and severely broadened resonances, the mean peak volume ratio between the corresponding minor and major peaks was 14% with an RMSD of 2%, indicating that 12% of the protein adopts this minor conformational state. A corresponding analysis for the C22V variant yielded a similar mean peak volume ratio. The 31 residues that exhibit resonance doubling form not only the majority of the active site, but also span much of the rest of the protein structure (Figure 3).

The largest chemical shift differences for the doubled resonances are seen for the non-proline residues in the 80′s loop which extends from the 3_10_ turn (Pro^78^–Ala^81^) to the start of the final β_5_ strand at Leu^97^. The residues in this loop provide the large proportion of interprotein interactions in the crystal structures of complexes formed by FKBP12 with calcineurin [48], mTOR [49] and tissue growth factor β_1_ receptor [50]. The 80′s loop has been noted to exhibit a wider diversity of conformations than the rest of the protein between different structures of FKBP12 as well as among structures of the various FKBP modules of the larger members of this protein family for which this loop has also been implicated in critical protein recognition interactions [51].

The resonance doubling extends beyond the 80′s loop into the first two residues of the β_5_ strand and the contiguous residues of the adjacent β_2_ and β_3α_ strands. The doubling also extends backwards along the chain into the 3_10_ turn and into the nearby α-helix bearing Trp^59^. Given the evidence for a single collective conformational transition underlying these resonance doublings as discussed below, the dynamic coupling between the 80′s loop and the α-helix may in part be mediated by the interactions of the large indole ring of Trp^59^ at the base of the active-site cleft. In addition, in the 0.92 Å X-ray structure of wild-type FKBP12 [30], the carbonyl oxygens of Tyr^80^ and Ala^81^ in the 3_10_ turn form a pair of hydrogen bonds to the amide hydrogens of Gly^58^ and Arg^57^ in the first turn of the α-helix respectively (the latter hydrogen bond is mediated by a crystallographic water molecule). Furthermore, the side chain of Ile^56^ is tightly packed against the aromatic ring of Tyr^82^.

The resonance doubling extends further to the beginning of the 50′s loop that surrounds the C-terminal end of the α-helix. In addition to the possibility of dynamical coupling mediated through the backbone interactions of the α-helix, the side chain of Glu^60^ forms hydrogen-bonding interactions with several peptide groups of the 50′s loop that are mediated by a conserved buried water molecule, as analysed in detail in the recent high-resolution crystallographic analysis of FKBP12 [30].

### Exchange spectroscopy of the slow conformational transition in FKBP12

A series of zz-exchange experiments were carried out to determine the rate of interchange between conformations giving rise to the doubling of resonances. Although most commonly used to measure ^1^H-^1^H cross relaxation rates between protons within 5–6 Å (in this case referred to as the NOESY experiment), this approach was initially introduced for measuring chemical exchange processes near the slow limit of exchange [52]. If the rate of chemical/conformational exchange (*k*_ex_) is comparable with the ^1^H longitudinal relaxation rate *R*_1_ or higher, the transition rate for a two-state process can be determined by fitting to the population equations:

$$\begin{array}{ccc} I_{\mathrm{AA}}\left(T\right) & = & P_{A}\left[P_{A}+P_{B}\cdot \exp\left(-k_{\mathrm{ex}}T\right)\right]\cdot \exp\left(-R_{1}T\right)\\ I_{\mathrm{BB}}\left(T\right) & = & P_{B}\left[P_{B}+P_{A}\cdot \exp\left(-k_{\mathrm{ex}}T\right)\right]\cdot \exp\left(-R_{1}T\right)\\ I_{\mathrm{AB}}\left(T\right) & = & I_{\mathrm{BA}}\left(T\right)=P_{A}P_{B}\left[1-\exp\left(-k_{\mathrm{ex}}T\right)\right]\cdot \exp\left(-R_{1}T\right) \end{array}$$

where *P*_A_ and *P*_B_ are the populations of the two states.

**Figure 4:**
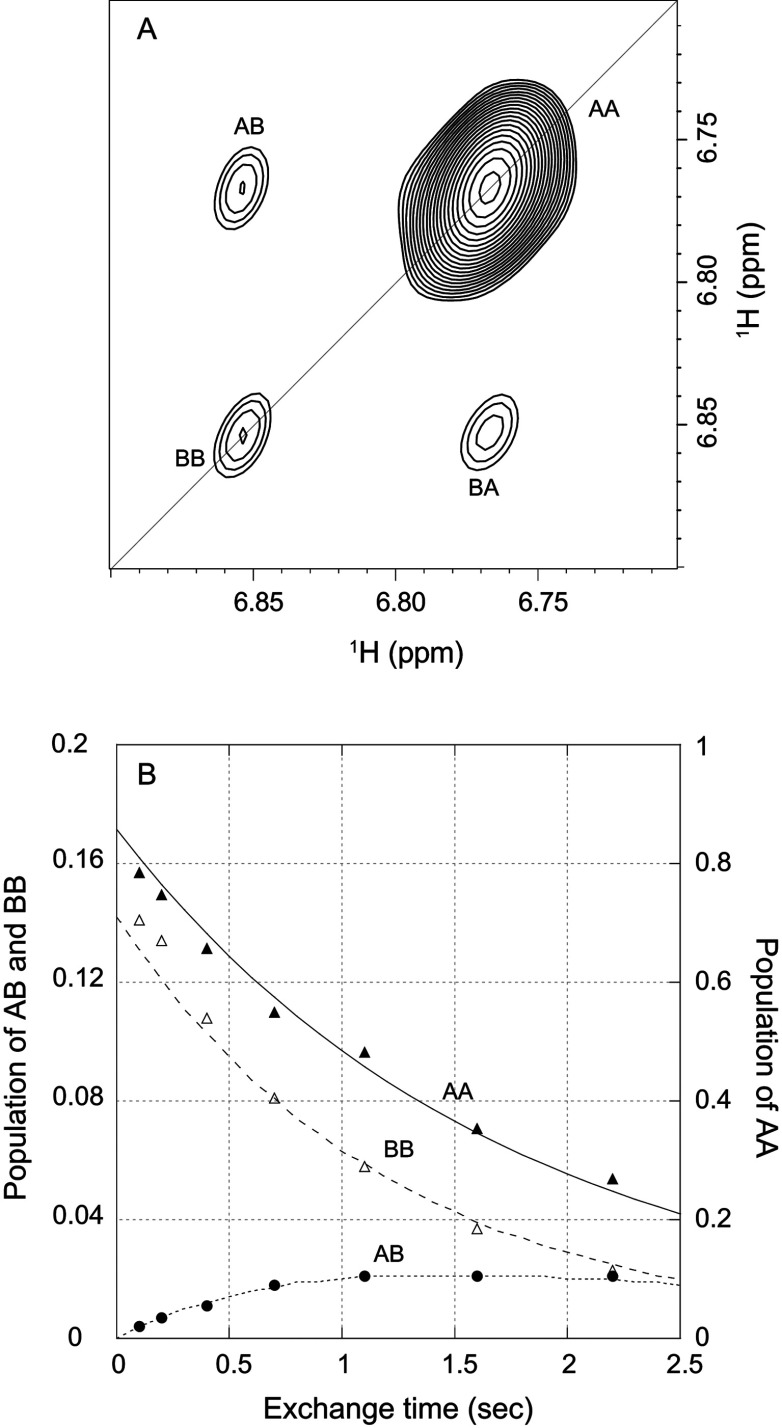
Kinetics of the slow conformational exchange in the C22V variant of FKBP12 at 43°C (**A**) zz-exchange diagonal and cross-peaks of the Trp^59^ indole H^Nϵ1^ in the major and minor conformational states at a mix time of 2.2 s. (**B**) Time course for the normalized peak intensities for the AA and BB diagonal peaks and for the AB cross-peak. The modest deviations in the predictions of the AA and BB diagonal peaks for early time points probably reflect a weak violation of equal *R*_1_ values for all states that is assumed in the model analysis which has a minimal effect on the derived conformational exchange constant.

Since sufficiently rapid transition rates were only observed at elevated temperatures, the moderately long collection times precluded the use of the less stable wild-type FKBP12, which precipitated under the conditions of these zz-exchange experiments despite the presence of both DTT and TCEP reducing agents. As illustrated for the indole side chain of Trp^59^ in a U-^2^H,^15^N-enriched sample of the C22V variant (Figure 4A), the two diagonal peaks arise from magnetization of the ^1^H nuclei that remain in the same conformational state at both the beginning and the end of the exchange mixing period, whereas the two off-diagonal cross-peaks arise from nuclei that change conformational state during that mixing period. When the intensity of these peaks are plotted against each other as a function of the exchange mixing period at 43°C, an exchange lifetime of 3.0 s is obtained (Figure 4B). A similar set of exchange measurements at 48°C yielded a conformational transition lifetime of 1.8 s, corresponding to an activation energy of 70 kJ/mol. A linear extrapolation to 25°C predicts a conformational transition lifetime of 20 s. The timeframe for this slow conformational exchange giving rise to resonance doublings is 100-fold longer than the global folding reaction of FKBP12 under similar conditions, with a rate of 4 s^−1^ at 25°C [53].

### Absence of prolyl isomerization in the slow conformational transition of FKBP12

For a number of proteins, slow conformational exchange between doubled resonances have been found to arise near sites in which the torsion angle of a disulfide bond flips from +90 ^o^ to −90 ^o^ [54,55] or an Xaa-Pro peptide bond undergoes a *cis*–*trans* isomerization [56,57]. Although FKBP12 lacks a disulfide linkage, the seven proline residues present the opportunity for a rate-limiting peptide isomerization in the native state. The most robust approach to characterizing the prolyl isomeric state in solution exploits the dependence of the C^β^ and C^γ^ chemical shifts on the equilibrium of the proline ring pucker distribution which, in turn, depends upon the *cis*–*trans* equilibrium of the peptide linkage [58]. Across a large number of proteins of known structure, the difference between the C^β^ and C^γ^ chemical shifts for *trans*-proline residues averaged 4.51 p.p.m. with an S.D. of 1.17 p.p.m. [59]. The corresponding values for *cis*-proline residues were 9.64 p.p.m. and 1.27 p.p.m. respectively.

**Figure 5:**
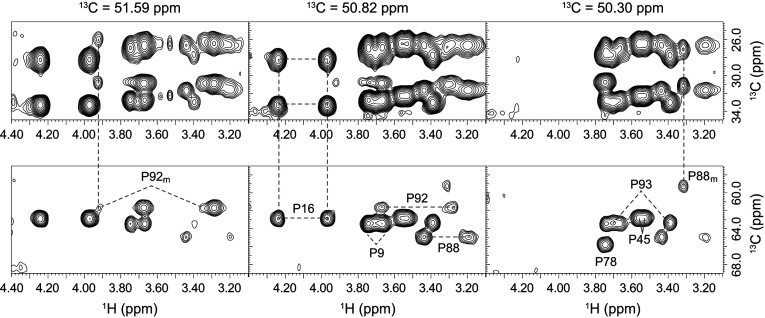
3D (H)CCH-TOCSY correlation for the seven proline residues of FKBP12 Spectral region for the proline ^13^C^β^ and ^13^C^γ^ (upper panels) and ^13^C^α^ (lower panels) resonances that are correlated to the intraresidue ^13^C^δ^ resonances at three frequencies within a 3D (H)CCH-TOCSY experiment. Complete connectivity patterns are observed for all seven proline residues in the major slow exchange state and for the resolved resonances of Pro^88^ and Pro^92^ in the minor slow exchange state. The small (4.0 p.p.m.) chemical shift differences for ^13^C^β^–^13^C^γ^ indicate that these two proline residues remain in a *trans*-peptide conformation in the minor slow exchange state.

A 2D CT-HSQC experiment resolved the ^1^H^δ^-^13^C^δ^ resonances of wild-type FKBP12 for the major slow exchange state of all seven proline residues and for the minor slow exchange state of Pro^88^ and Pro^92^ (Supplementary Figure S1 at http://www.biochemj.org/bj/453/bj4530371add.htm). 2D planes from a 3D (H)CCH-TOCSY experiment yielded connectivity patterns linking the ^13^C^δ^ resonance of each proline to the intraresidue C^α^, C^β^ and C^γ^ resonances (Figure 5). In every case, the difference between the C^β^ and C^γ^ chemical shifts is close to 4.5 p.p.m. (4.0 p.p.m. for both Pro^88^ and Pro^92^ in the minor state), indicating that all proline residues remain in a *trans* conformation in both the major and minor slow exchange states. Particularly noteworthy is the 5.7 p.p.m. upfield shift of the Pro^88^ C^α^ resonance that occurs upon transition into the minor slow exchange state (Figure 5). Similarly large changes in chemical shift occur for the ^15^N and ^13^C^α^ resonances of Gly^89^. Chemical shift analysis of the backbone resonances with the TALOS+ algorithm [60] predicts Φ and Ψ torsion angles of (88, −7) for Gly^89^ in the major slow exchange state {X-ray structure [30] yields (103, −28)}, while torsion angles of (−59, −27) are predicted for the minor slow exchange state. These results suggest that the switch from a positive to a negative Φ angle for Gly^89^ constitutes a major aspect of the structural transition underlying the resonance doubling behaviour of FKBP12.

### Magnetic field-dependence of ^15^N NMR relaxation in characterizing conformational exchange-induced line-broadening in FKBP12

**Figure 6:**
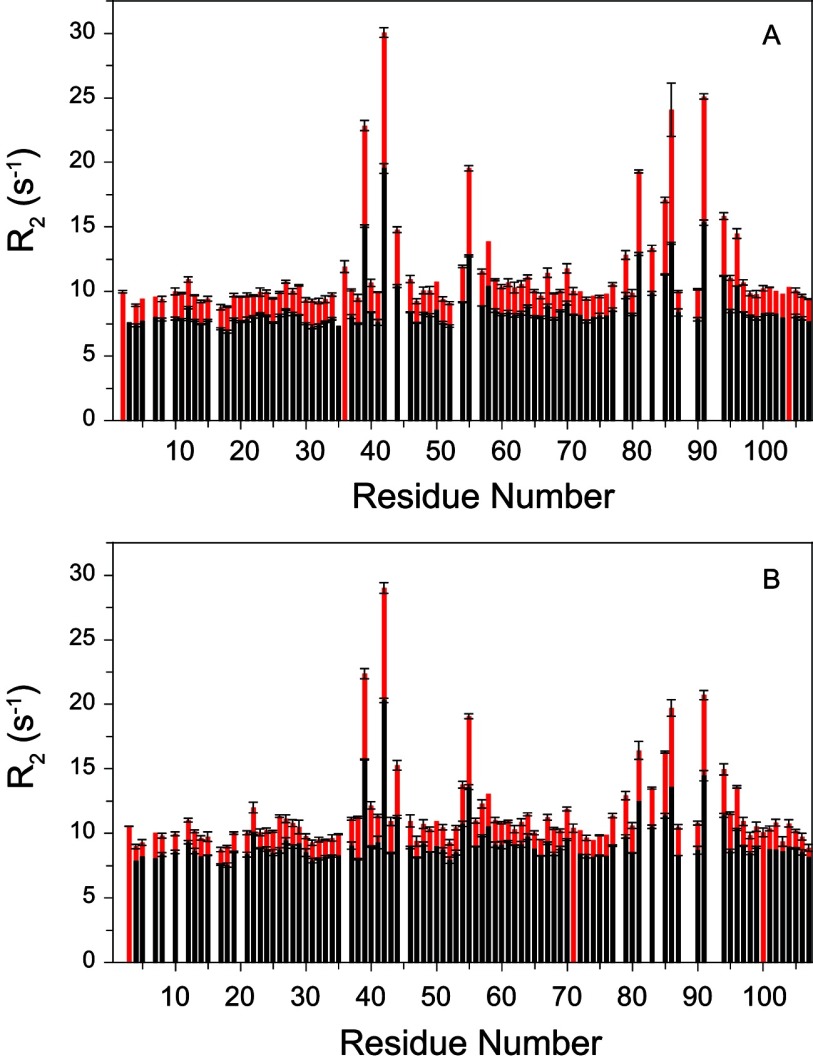
Magnetic field-dependence of the ^15^N transverse relaxation rates for the resonances of the major slow exchange conformation in the wild-type and C22V variant of FKBP12 at 25°C (**A**) *R*_2_ values for the wild-type FKBP12 at 600 MHz ^1^H (black) and 800 MHz ^1^H (red). Residues not undergoing conformational exchange-dependent line-broadening exhibit field-dependent increases in *R*_2_ values of ~23%. (**B**) *R*_2_ values for the C22V variant of FKBP12 at 600 MHz ^1^H (black) and 800 MHz ^1^H (red). Relaxation data are not reported for the severely broadened resonances of Tyr^82^ (conformational exchange broadening), Ala^84^ (amide hydrogen exchange broadening) and Gly^89^ (both).

The timeframe for the slow conformational exchange differs by five orders of magnitude from that for the fast limit line-broadening transition (τ≈100 μs) [13,14] discussed above. Nevertheless, there are noteworthy similarities between the set of residues involved in these two classes of transitions. For conformational transitions in the fast limit regime of NMR exchange broadening (τ≈20–500 μs), the increase in the transverse relaxation rate *R*_2_ arising from these conformational dynamics scales as the square of the magnetic field as illustrated for the major conformer ^15^N resonances of the wild-type and the C22V variant of FKBP12 at 14.1 T (^1^H 600 MHz) and 18.8 T (^1^H 800 MHz) (Figure 6). The majority of residues yield similar *R*_2_ values near 8 s^−1^ at 600 MHz and 10 s^−1^ at 800 MHz, which combined with the corresponding *R*_1_ longitudinal relaxation times and NOE values (Supplementary Figure S2 at http://www.biochemj.org/bj/453/bj4530371add.htm), are consistent with an isotropically tumbling global correlation time of 5.8 ns. A protein concentration of 1 mM was used in these measurements, as we observed an appreciable increase in the *R*_2_ values for higher concentrations.

As expected, the residues of wild-type FKBP12 that exhibit a significant magnetic field-dependence in their *R*_2_ values closely correspond to the residues for which Brath and Akke [14] reported ^15^N relaxation dispersion curves under similar experimental conditions. Consistent with fast limit exchange dynamics, the exchange contribution to the present *R*_2_ relaxation rates closely correlate (*r*=0.979) with the Φ_ex_ values that Brath and Akke derived from their relaxation dispersion analysis (Supplementary Figure S3 at http://www.biochemj.org/bj/453/bj4530371add.htm).

**Figure 7:**
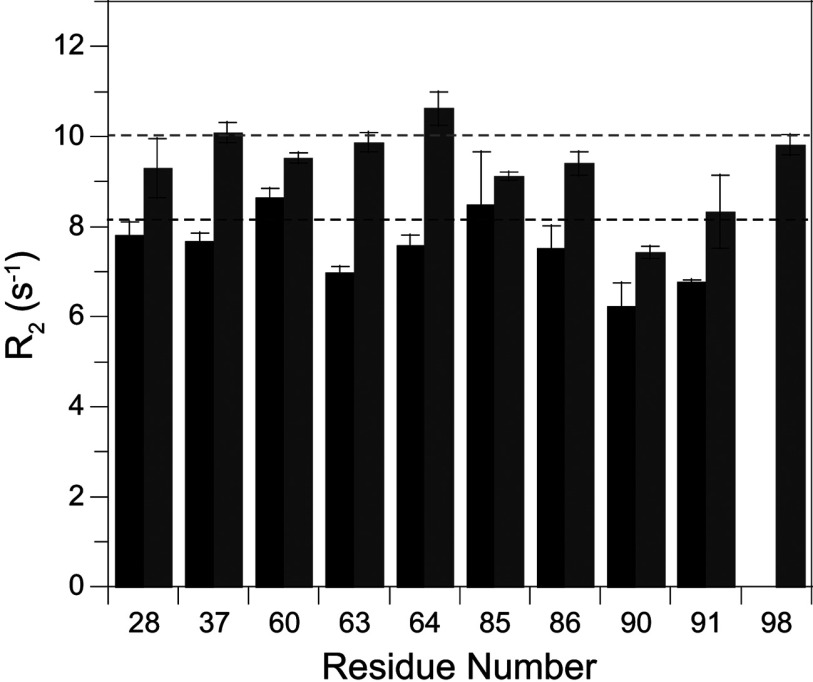
Magnetic field-dependence of the ^15^N transverse relaxation rates for the resolved resonances of the minor slow exchange conformation in the wild-type FKBP12 at 25°C For the residues that give rise to fully resolved resonances for the minor slow exchange conformation, the *R*_2_ relaxation rates at 600 MHz ^1^H (black) and 800 MHz ^1^H (grey) do not significantly exceed the average *R*_2_ relaxation value observed for the residues which do not exhibit conformational exchange-dependent line-broadening (broken lines). The lower *R*_2_ values observed for residues 90 and 91 correlate with comparably lower *R*_1_ and NOE values at these sites (see Supplementary Figure S2 at http://www.biochemj.org/bj/453/bj4530371add.htm), consistent with internal mobility in the picosecond–nanosecond timeframe for the tip of the 80′s loop.

A number of the residues that exhibit conformational exchange line-broadening also give rise to resonance doubling. The differences in ^15^N chemical shift between the major and minor peaks for these residues do not exhibit a strong correlation with the magnitude of the field-dependent ^15^N *R*_2_ line-broadening as could be anticipated if the conformations of the minor states for both the slow and fast transitions were to be structurally similar. A more definitive demonstration of a qualitative distinction between the properties of the slow exchanging (τ≈20 s at 25°C) major and minor species comes from the *R*_2_ relaxation rates for the minor species resonances that are sufficiently resolved to enable reliable peak integration. All of these well-resolved minor slow exchange state resonances have *R*_2_ values that are quite similar to the *R*_2_ values for residues in the major slow exchange state that do not exhibit any conformational exchange broadening (Figure 7, and Supplementary Figure S4 at http://www.biochemj.org/bj/453/bj4530371add.htm). Regarding the spatial extent of this correlation, only half of the residues having a well resolved resonance in the minor slow exchange state also exhibit line-broadening in the major slow exchange state, and all of the residues exhibiting both characteristics lie within the 80′s loop. Thus, at minimum, the slow transition to the minor state conformation results in the quenching of the 10^5^-fold more rapid line-broadening dynamics in the 80′s loop.

The conformational exchange broadening behaviour of the major and minor slow exchange states of the C22V variant closely follows that of the wild-type protein, indicating that the valine substitution has a minimal effect on either of these two distinct dynamic processes. Given the superior chemical stability of the C22V variant, the additional mutations considered below were all introduced into this background.

### Modulation of FKBP12 conformational dynamics by mutational studies

As an X-ray structure determination of the yeast (*S. cerevisiae*) FKBP12 bound to FK506 has been reported [17], we carried out similar NMR measurements on the unligated form of that protein with the intent of gaining structural insight into these two classes of conformational transitions. Immediately apparent was the fact that the set of minor peaks in the yeast protein were attenuated 4-fold from their relative intensity in the human protein (Supplementary Figure S5 at http://www.biochemj.org/bj/453/bj4530371add.htm). The yeast and human FKBP12 sequences are reasonably similar within the 80′s loop which exhibits the largest differential shifts between the major and minor slow exchange states. One noteworthy difference is the presence of phenylalanine in the yeast protein at position 87 (histidine in the human enzyme), which in the crystal structure sticks out into the substrate-binding pocket. When the H87F mutation was introduced into the human FKBP12 sequence, the population of the minor slowing exchanging state was again attenuated ~4-fold relative to the parental human sequence.

Given that residue 87 plays a significant role in modulating the population of the minor slow exchange state, we analysed the H87V variant of the human protein. Navia and colleagues have reported the X-ray structures of the H87V and H87V+R42K variants of FKBP12 bound to FK506 and deposited the co-ordinates of the double mutant in the PDB (PDB code 1BKF) [61]. They reported that the C^α^ co-ordinates of the H87V variant have an RMSD of 0.277 Å with respect to their reference native structure. Minimal structural distortion occurs around the site of mutation.

**Figure 8:**
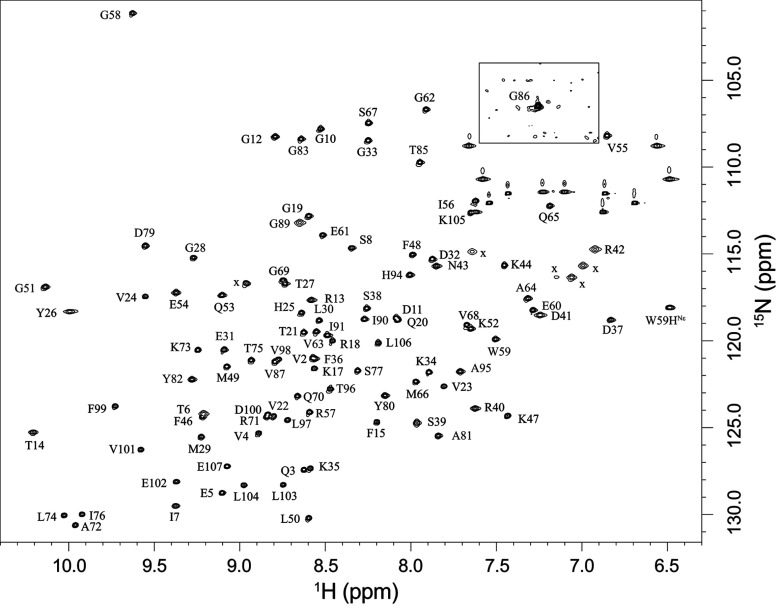
^1^H-^15^N 2D NMR correlation spectrum of U-^2^H,^15^N-enriched H87V variant of FKBP12 Spectral region surrounding the resonance for Gly^86^ is plotted at an 8-fold lower contour to illustrate the absence of a minor peak arising from slow conformational exchange. Folded side-chain resonances are indicated with x.

We found that substitution of valine at position 87 dramatically suppresses the minor state of the slow exchange transition. No evidence for any minor state resonance was observed for any of the 31 residues that exhibit doubling in wild-type FKBP12, indicating an upper limit of ~0.2% for the minor state (Figure 8). Given the 12% population of the minor state in wild-type FKBP12, the H87V substitution shifts the equilibrium of the slow exchange transition at least 60-fold or by more than 10 kJ/mol. Such a large effect on the equilibrium of this transition is consistent with the H87V mutation being closely connected to the primary site of structural alteration from which the chemical shift differences discussed above appear to be around the Pro^88^–Gly^89^ linkage. On the other hand, as illustrated in the mutational analysis of *cis*–*trans*-proline equilibria in staphylococcal nuclease [62], substitutions well removed from the site of proline isomerization can significantly alter the *cis*/*trans* ratio. The ratio for the major and minor slow exchange conformations of FKBP12 is unchanged from pH 5.5 to pH 9.0 (p*K*_a_ of His^87^ is 5.92 [47]), indicating that the effect of the valine substitution does not arise indirectly from eliminating the ionization of the imidazole side chain.

**Figure 9:**
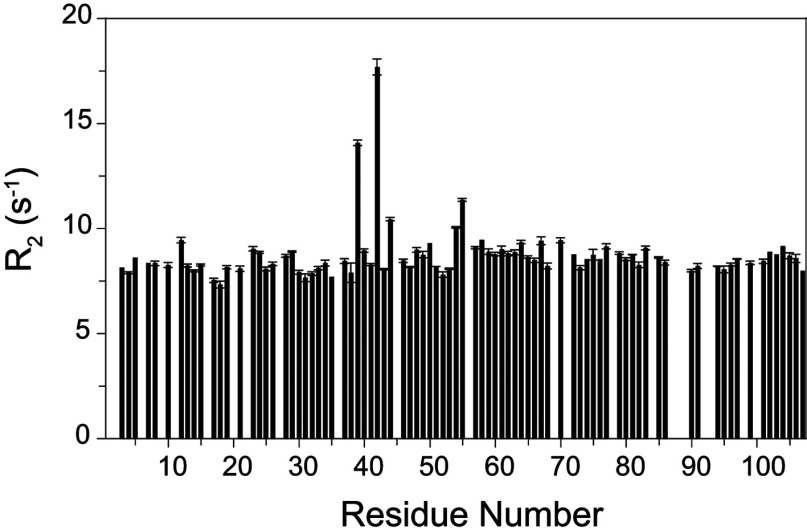
^15^N transverse relaxation rates for the H87V variant of FKBP12 at 25°C *R*_2_ values for the C22V/H87V variant of FKBP12 at 600 MHz ^1^H. As compared with the wild-type and C22V variant (Figure 5), conformational exchange-dependent line-broadening is fully quenched in the residues of the 80′s loop, whereas line-broadening in the α-helix and preceding 50′s loop is reduced. In contrast, line-broadening in the 40′s loop is similar to that of the wild-type and C22V variant FKBP12 proteins.

The *R*_1_ and NOE values for the H87V variant are quite similar to those for the major species of the wild-type protein, indicating similar dynamics in the picosecond–nanosecond timeframe. In contrast, the *R*_2_ values for the H87V variant differ markedly. The conformational broadening for the residues in the 80′s loop and preceding 3_10_ helix was suppressed completely, whereas the broadened resonances in the 40′s loop were seemingly unaffected (Figure 9). An intermediate behaviour was seen for residues near the beginning of the α-helix. For Glu^54^, Val^55^ and Gly^58^, the conformational broadening contribution decreased ~35% in comparing the H87V variant with either the wild-type protein or the K44V variant discussed below. The decoupling of the conformational broadening dynamics between different regions of the protein induced by the H87V mutation clearly indicates that the apparent global character of the motions in this timeframe proposed in previous relaxation studies [13,14,16] is coincidental.

A complimentary behaviour was observed when valine was introduced at residue 44. The line-broadening from conformational exchange at residues 39, 42 and 44 decreased 3-fold, whereas none of the other amides of the protein were affected (Supplementary Figure S6 at http://www.biochemj.org/bj/453/bj4530371add.htm). More strikingly, none of the resonance doublings exhibited by the wild-type protein were affected by the K44V mutation, including that for the immediately adjacent Ser^38^. Noteworthy is the close similarity between the resonances for the major exchange state in the ^1^H-^15^N 2D correlation spectrum of the K44V variant (Supplementary Figure S7 at http://www.biochemj.org/bj/453/bj4530371add.htm) and those of the H87V variant (Figure 8), indicating how precisely the structure is preserved upon these mutations.

Our NMR relaxation studies of FKBP12 variants demonstrate that the conformational broadening dynamics described previously appear to involve at least three distinct processes centred on the 80′s loop, the 50′s loop + α-helix and the 40′s loop respectively. Motion in the 40′s loop, as indicated by line-broadening at Ser^39^, Arg^42^ and Lys^44^, is dynamically uncoupled from other significant transitions of the protein in this timeframe. A structural basis for the decoupling of these conformational dynamics might be anticipated from the substantial distance between the residues of the 40′s loop and either those of the 80′s loop or of the 50′s loop and α-helix. On the other hand, line-broadening in the 80′s loop is efficiently suppressed by the modest structural alteration of the Val^87^ substitution, whereas the conformational dynamics of the 50′s loop and the adjacent α-helix are also significantly affected. As considered in the discussion of dynamical coupling for the slow exchange transition above, the direct interactions between residues of the 3_10_ turn at the start of the 80′s loop and Ile^56^, Arg^57^ and Gly^58^ at the start of the α-helix could potentially mediate coupling between these two regions for conformational transition occurring within the line-broadening time regime (τ≈100 μs).

The H87V-induced suppression of line-broadening for many of the residues surrounding the active site does not technically demonstrate that the underlying conformational transition has been suppressed. Formally, the valine substitution could have accelerated the rate of that transition to above ~50000 s^−1^ so that the line-broadening effect is eliminated. In part due to the attendant elimination of the widespread resonance doubling, a direct suppression of the underlying conformational transition provides a considerably more plausible interpretation. The peptidylprolyl isomerase activity of FKBP12 is unaffected by the substitution of valine at position 87 [61]. This lack of effect on the catalytic activity for a mutation that suppresses the conformational broadening behaviour for much of the active site appears to be inconsistent with the earlier NMR relaxation analysis that ascribed this line-broadening to a sampling of the transition state conformation by the unligated enzyme [14].

At least for residues in the 80′s loop that exhibit resonance doubling as well as conformational exchange broadening for the major slow exchange form, the conformational exchange broadening is suppressed for the minor slow exchange form. A model for rationalizing this behaviour posits that the minor slow exchange state is kinetically accessed via the minor form of the ~100 μs line-broadening transition of the 80′s loop. Substitution of valine at residue 87 inhibits this ~100 μs transition to the minor form which, in turn, precludes the larger scale transition responsible for the extensive resonance doubling.

### Crystal structure for the H87V variant of FKBP12

To gain further structural insight into how the H87V substitution so dramatically alters the dynamical properties of the protein, we carried out a crystallographic analysis of this variant protein at 1.70 Å resolution (Table 1, crystallographic summary). Overall, there is a 0.25 Å C^α^ RMSD between this structure and the 0.92 Å resolution structure of the wild-type protein (PDB code 2PPN [30]). In particular, the minimal differences in structure applies to the region surrounding residue 22 for which the wild-type cysteine residue is replaced by valine. The only appreciable shift in heavy atom position at this site is for Leu^103^ C^δ1^ which moves 0.6 Å away from the newly introduced Val^22^ C^γ1^ methyl group.

**Table 1:** Crystallographic data collection, refinement and model details (PDB code 4IPX) Values in parentheses are for the highest resolution shell. MPD, 2-methyl-2,4-pentanediol

Paramater	Value
Data collection	
Resolution range (Å)	35–1.70 (1.78–1.70)
Number of unique reflections	10996
Redundancy	3.2 (3.4)
Completeness (%)	93.7 (93.6)
Average *I*/σ(*I*)	29.4 (6.3)
*R*_merge_ (%)	9.7 (29.6)
Refinement	
Resolution limits (Å)	35–1.70
Number of reflections	10996
*R*_work_ (%)	20.8
*R*_free_ (%)	21.8
Non-hydrogen atoms	
Protein	830
MPD	16
Water	127
Average *B*-factor (Å^2^)	
All atoms	16.8
Solvent	29.5
Geometry	
RMSD bond length (Å)	0.003
RMSD bond angle (°)	0.815

**Figure 10:**
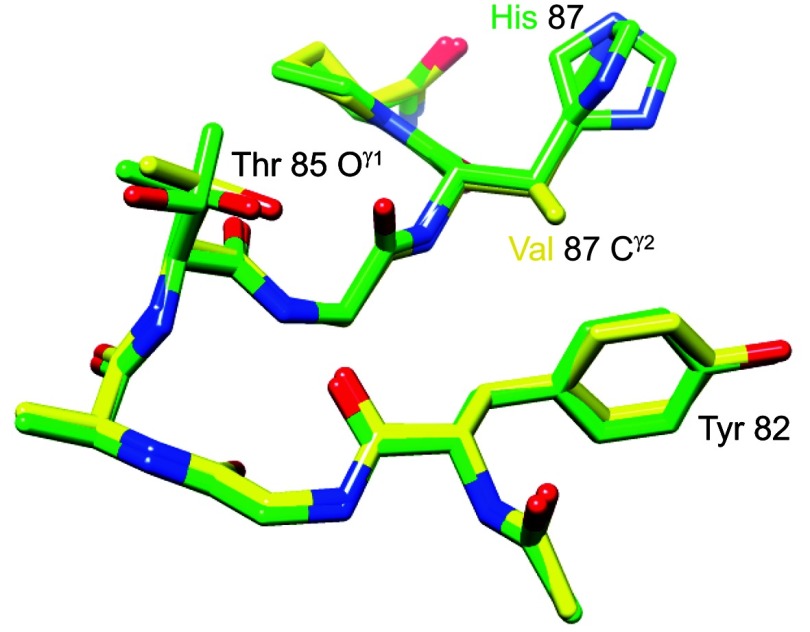
Superimposition of region surrounding residue 87 from the 1.70 Å resolution structure of the H87V variant of FKBP12 and the 0.92 Å resolution wild-type structure The carbon atoms of the H87V variant are indicated in yellow, whereas those of the wild-type protein (including dual conformers for the side chains of Thr^85^ and His^87^) are indicated in green. The C^γ2^ atom of Val^87^ is 3.7 Å from both the C^γ^ and C^δ1^ atoms of Tyr^82^.

**Figure 11:**
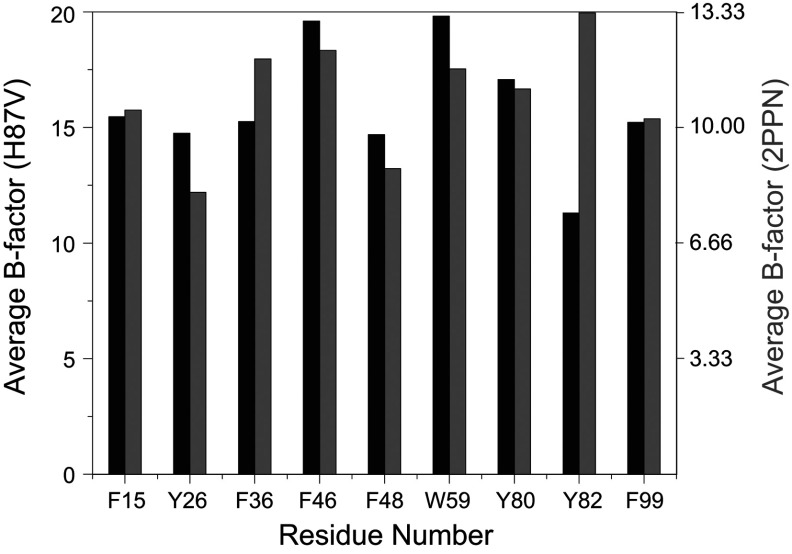
Average aromatic ring atom crystallographic *B*-factors for the residues of the H87V variant and wild-type FKBP12 The average *B*-factor for the aromatic ring atoms is 33% smaller in the higher-resolution structure of the wild-type [30]. Although for the other aromatic residues the ring *B*-factors correlate quite closely for the H87V (black) and wild-type (grey) structures, the relative *B*-factor for the ring of Tyr^82^ is markedly decreased in the H87V structure.

Beyond the changes in covalent structure caused by substitution of valine for histidine at residue 87, there is strikingly little alteration within this region of the protein (Figure 10). The C^γ1^ atom of Val^87^ lies upon the histidine C^γ^ atom of the wild-type structure, while the valine C^γ2^ projects toward the face of the aromatic ring of Tyr^82^ which is not displaced but appears to become more constrained. Of the nine aromatic phenylalanine, tyrosine and tryptophan residues in FKBP12, the ring atoms of Tyr^82^ have the highest average crystallographic *B*-factor in the wild-type structure, but the lowest average *B*-factor in the H87V structure (Figure 11). This rigidification of the Tyr^82^ side-chain mobility is consistent with the reduced conformational dynamics of the Tyr^82^ backbone that is indicated by the marked narrowing of the amide resonance of this residue in the H87V NMR spectra.

The H87V mutation appears to lock the protein in a conformation closely similar to the major form of wild-type FKBP12. The modest structural changes induced by the substitution of valine for histidine at residue 87 are sufficient to suppress the transition to the minor state of the spatially extensive slow exchange process at least 60-fold. This structurally conservative mutation also serves to suppress the much more rapid conformational line-broadening dynamics in the 80′s loop and partially suppress mobility in this time regime in the region surrounding the start of the α-helix. Further structural analysis of conformational plasticity in the region surrounding the 80′s loop may provide useful insight into the wide range of intermolecular recognition interactions mediated by this loop for the various members of the FKBP family of proteins.

## Online data

Supplementary data
