# Supplementary material for: Analysing the visible conformational substates of the FK506-binding protein FKBP12
Source: Biochem J. 2013 Jul 12;453(Pt 3):371–80. doi: 10.1042/BJ20130276 (PMC3727217; doi:10.1042/BJ20130276)
Supplement: Supplementary data [file bj4530371add.pdf]

BJ Biomolecules

Biochem. J. (2013) **453**, 371–380 (Printed in Great Britain) doi:10.1042/BJ20130276

online data

SUPPLEMENTARY ONLINE DATA

Analysing the visible conformational substates of the FK506-binding protein FKBP12

Sourajit M. MUSTAFI*, Hui CHEN*[1], Hongmin LI*†, David M. LeMASTER*† and Griselda HERNÁNDEZ*†[2]

*Wadsworth Center, New York State Department of Health, Empire State Plaza, Albany, NY 12201, U.S.A., and †Department of Biomedical Sciences, School of Public Health, University at Albany - SUNY, Empire State Plaza, Albany, NY 12201, U.S.A.

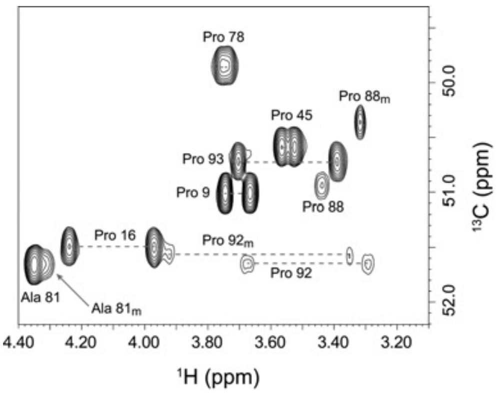

Figure S1 Proline $^1H^\delta$–$^{13}C^\delta$ cross-peaks in the 2D CT-HSQC NMR correlation spectrum of wild-type FKBP12

Resolved resonances were observed for the major slow exchange state for all seven proline residues as well as for Pro^{88} and Pro^{92} in the minor slow exchange state. Owing to conformational exchange line-broadening, the major state cross-peaks for Pro^{88} and Pro^{92} are strongly attenuated, yielding intensities that are similar to those observed for the minor state resonances of these residues. Indeed, the resonance for the upfield $^1H^\delta$ of Pro^{88} at 3.198 p.p.m. is only observable at a lower contour level, reflecting even more severe line-broadening at the high (900 MHz ^{1}H) magnetic field used for this measurement.

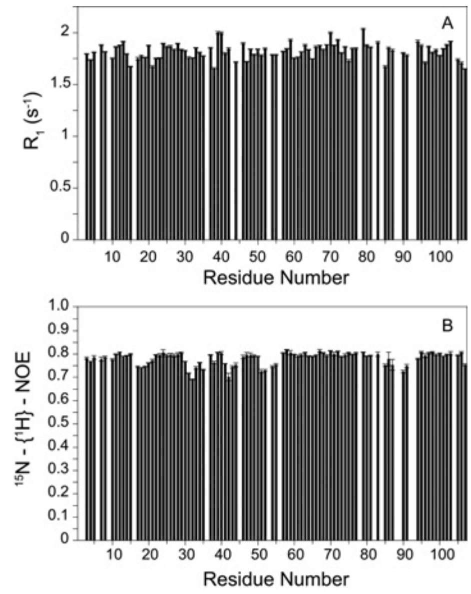

Figure S2 ^{15}N longitudinal relaxation rates (R_1) and heteronuclear NOE values for the resonances of the major slow exchange conformation in wild-type FKBP12 for 14.1 T (600 MHz ^{1}H) at 25°C

Relaxation data are not reported for the severely broadened resonances of Tyr^{82} (conformational exchange broadening), Ala^{84} (amide hydrogen exchange broadening) and Gly^{89} (both).

Biochemical Journal www.biochemj.org

[1] Present address: Life Sciences Institute, University of Michigan, Ann Arbor, MI 48109, U.S.A.
[2] To whom correspondence should be addressed (email griselda@wadsworth.org).

Co-ordinates for the reported protein structure of FKBP12 have been deposited in the PDB under code 4IPX.

Chemical shift data have been deposited in the BMRDB (Biological Magnetic Resonance Data Bank) under accession numbers 19240 for the major form and 19241 for the minor form of FKBP12.

© 2013 The Author(s)

The author(s) has paid for this article to be freely available under the terms of the Creative Commons Attribution Licence (CC-BY) (http://creativecommons.org/licenses/by/3.0/) which permits unrestricted use, distribution and reproduction in any medium, provided the original work is properly cited.

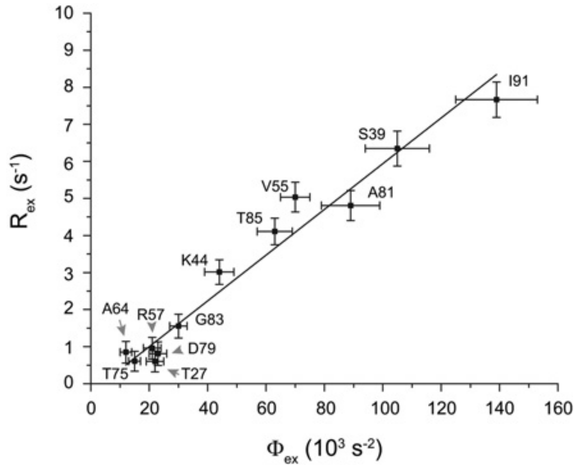

Figure S3 Correlation between the exchange contribution to CPMG-based R_2 relaxation rates of wild-type FKBP12 at 25 °C and exchange contributions derived from relaxation dispersion measurements obtained under similar experimental conditions [1]

In the fast exchange limit analysis of relaxation dispersion data for a two-state process, $\Phi_{ex} = \Delta\omega^2 P_A(1 - P_A) = R_{ex}(k_{ex}^2 + \omega_{eff}^2)/k_{ex}$, where $\Delta\omega$ is the chemical shift difference between the two conformations, P_A is the population of the major conformation, R_{ex} is the exchange contribution to R_2, k_{ex} is the conformational exchange rate and ω_{eff} is the effective spin-lock field. The CPMG-based R_{ex} estimates were derived from extended Lipari–Szabo analysis [2,3] of the ^{15}N R_1, R_2 and NOE relaxation data (Figure 6 of the main text and Figure S2) as implemented with Fast Modelfree software [4].

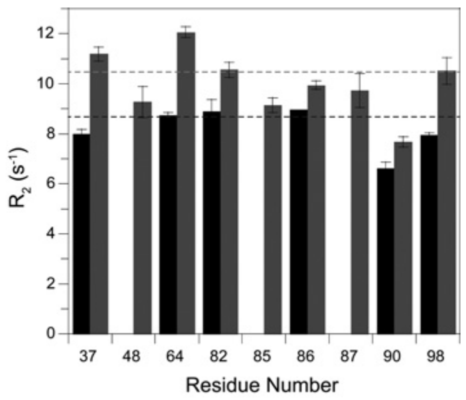

Figure S4 Magnetic field dependence of the ^{15}N transverse relaxation rates for the resolved resonances of the minor slow exchange conformation in the C22V variant of FKBP12 at 25 °C

For the residues that give rise to fully resolved resonances for the minor slow exchange conformation, the R_2 relaxation rates at 600 MHz ^1H (black) and 800 MHz ^1H (grey) do not significantly exceed the average R_2 relaxation value observed for the residues which do not exhibit conformational exchange-dependent line-broadening (broken lines). The lower R_2 values observed for residue 90 correlate with comparably lower R_1 and NOE values at this site, consistent with internal mobility in the picosecond–nanosecond timeframe for the tip of the 80′s loop.

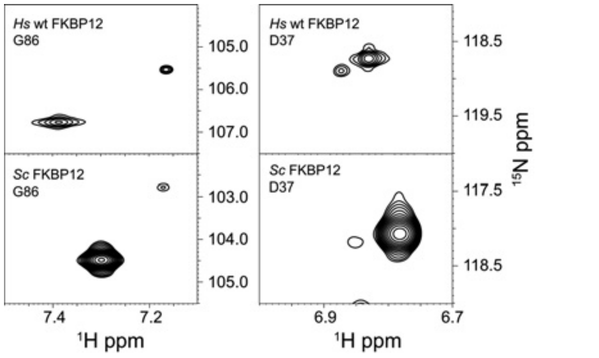

Figure S5 Resonance doubling in the ^1H-^{15}N 2D NMR correlation spectrum of *S. cerevisiae* FKBP12

As illustrated for Gly[86] and Asp[37], the C48L variant of *S. cerevisiae* FKBP12 exhibits minor conformer resonances quite similar to those observed for wild-type human FKBP12, although the relative intensity is reduced ∼4-fold.

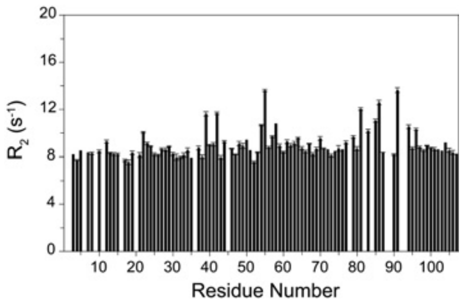

Figure S6 ^{15}N transverse relaxation rates for the K44V variant of FKBP12 at 25 °C

R_2 values for the C22V/K44V variant of FKBP12 at 600 MHz ^1H. As compared with the wild-type and C22V variant, conformational exchange-dependent line-broadening for residues of the 40′s loop are reduced approximately 3-fold, whereas the conformational exchange-dependent line-broadening for other residues in the protein appear to be unaffected.

© 2013 The Author(s)
The author(s) has paid for this article to be freely available under the terms of the Creative Commons Attribution Licence (CC-BY) (http://creativecommons.org/licenses/by/3.0/) which permits unrestricted use, distribution and reproduction in any medium, provided the original work is properly cited.

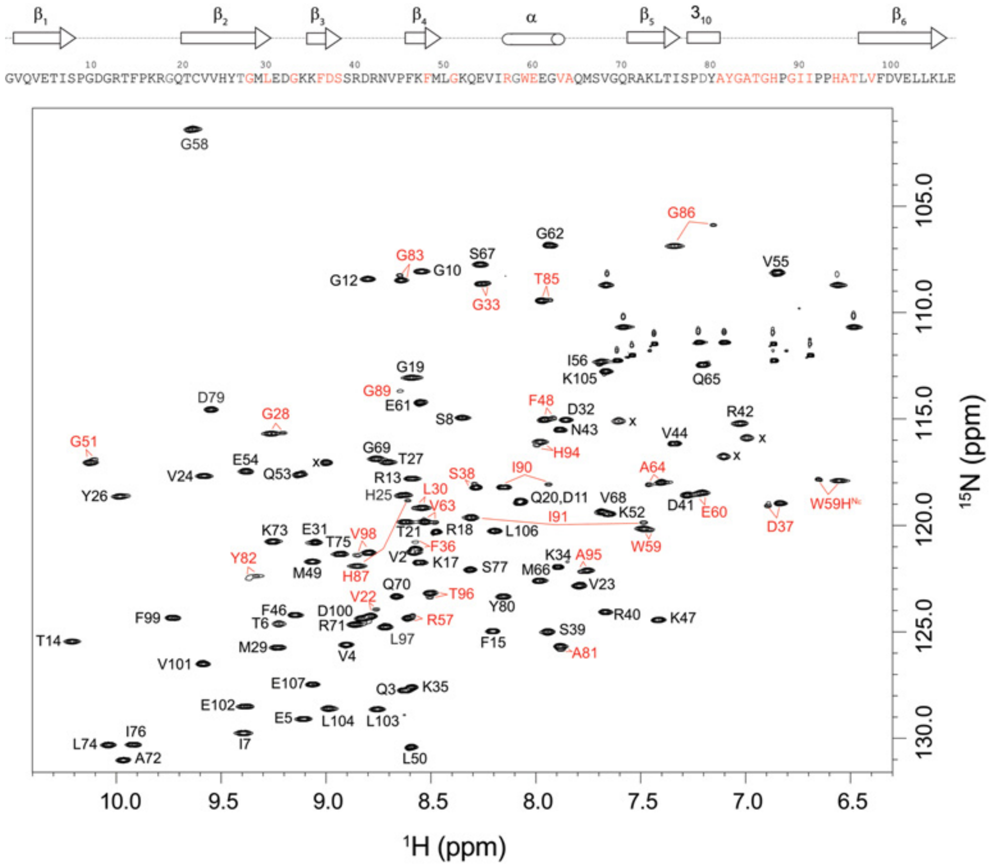

Figure S7 **^{1}H-^{15}N 2D NMR correlation spectrum of U-^{15}N-enriched C22V/K44V variant of FKBP12**

Residues exhibiting resolved resonances for the minor slow exchange conformation are indicated in red. As data collection was carried out on an ^{15}N-enriched sample of the K44V variant without perdeuteration, the spectral resolution is modestly reduced, relative to that of the wild-type spectrum (Figure 2 of the main text). Ala^{84} and Gly^{89} exhibit the most rapid amide hydrogen exchange in the protein, resulting in severe broadening in the ^{1}H dimension at pH 6.5. Folded side-chain resonances are indicated with x.

REFERENCES

1 Brath, U. and Akke, M. (2009) Differential responses of the backbone and side-chain conformational dynamics in FKBP12 upon binding the transition-state analog FK506: implications for transition-state stabilization and target protein recognition. J. Mol. Biol. **387**, 233–244

2 Lipari, G. and Szabo, A. (1982) Model-free approach to the interpretation of nuclear magnetic resonance relaxation in macromolecules. 1. Theory and range of validity. J. Am. Chem. Soc. **104**, 4546–4559

3 Clore, G. M., Szabo, A., Bax, A., Kay, L. E., Driscoll, P. C. and Gronenborn, A. M. (1990) Deviations from the simple two-parameter model-free approach to the interpretation of nitrogen-15 nuclear relaxation of proteins. J. Am. Chem. Soc. **112**, 4989–4991

4 Cole, R. and Loria, J. P. (2003) FAST-Modelfree: a program for rapid automated analysis of solution NMR spin-relaxation data. J. Biomol. NMR **26**, 203–213

Received 20 February 2013/15 May 2013; accepted 21 May 2013
Published as BJ Immediate Publication 21 May 2013, doi:10.1042/BJ20130276

© 2013 The Author(s)
The author(s) has paid for this article to be freely available under the terms of the Creative Commons Attribution Licence (CC-BY) (http://creativecommons.org/licenses/by/3.0/) which permits unrestricted use, distribution and reproduction in any medium, provided the original work is properly cited.
